# Sound-mapping a coniferous forest—Perspectives for biodiversity monitoring and noise mitigation

**DOI:** 10.1371/journal.pone.0189843

**Published:** 2018-01-10

**Authors:** Anthony Turner, Michael Fischer, Joseph Tzanopoulos

**Affiliations:** 1 Durrell Institute of Conservation and Ecology, School of Anthropology and Conservation, University of Kent, Canterbury, Kent, United Kingdom; 2 Kent’s Interdisciplinary Centre for Spatial Studies (KISS), University of Kent, Canterbury, Kent, United Kingdom; Urbino University, ITALY

## Abstract

Acoustic diversity indices have been proposed as low-cost biodiversity monitoring tools. The acoustic diversity of a soundscape can be indicative of the richness of an acoustic community and the structural/vegetation characteristics of a habitat. There is a need to apply these methods to landscapes that are ecologically and/or economically important. We investigate the relationship between the acoustic properties of a coniferous forest with stand-age and structure. We sampled a 73 point grid in part of the UK’s largest man-made lowland coniferous plantation forest, covering a 320ha mosaic of different aged stands. Forest stands ranged from 0–85 years old providing an age-gradient. Short soundscape recordings were collected from each grid point on multiple mornings (between 6am-11am) to capture the dawn chorus. We repeated the study during July/August in 2014 and again in 2015. Five acoustic indices were calculated for a total of 889 two minute samples. Moderate relationships between acoustic diversity with forest stand-age and vegetation characteristics (canopy height; canopy cover) were observed. Ordinations suggest that as structural complexity and forest age increases, the higher frequency bands (4-10KHz) become more represented in the soundscape. A strong linear relationship was observed between distance to the nearest road and the ratio of anthropogenic noise to biological sounds within the soundscape. Similar acoustic patterns were observed in both years, though acoustic diversity was generally lower in 2014, which was likely due to differences in wind conditions between years. Our results suggest that developing these relatively low-cost acoustic monitoring methods to inform adaptive management of production landscapes, may lead to improved biodiversity monitoring. The methods may also prove useful for modelling road noise, landscape planning and noise mitigation.

## Introduction

The global landscape is increasingly being modified by anthropogenic activities. Unsustainable forestry and intensive agricultural practises, and the resulting habitat fragmentation, degradation and loss, are recognised as one of the greatest threats to biodiversity worldwide [1,2]. This has led to the development of commodity certification schemes, eco-labelling and agri-environment schemes that aim to redress the balance between economic viability, social equality and environmental destruction[3]. Integral to the success of these schemes is the effective monitoring of biodiversity to enable comparative assessments within and between scheme participants. However, due to the complexities associated with monitoring biodiversity, in terms of selecting suitable methods, monetary costs and time-constraints, few schemes offer quantitative evidence-based protocols on how to monitor biodiversity within participating landscapes [4,5]. Without suitable monitoring of biodiversity, the efficiency of management practises can be misinterpreted and misunderstood [5,6].

Selecting suitable indicators for monitoring biodiversity is notoriously difficult. The term ‘biodiversity’ can be used to describe many aspects of nature, including genetic diversity, species diversity, species distributions, community composition, functional diversity, the diversity of habitats within a landscape, landscapes within a region and regions globally [7]. Species-richness and diversity and other associated composite indices are perhaps the most traditional and often considered the simplest way to describe biodiversity [8]. As such, the use of taxonomic communities as indicators (such as birds, beetles, ants or plants) can be considered cost-effective in some circumstances [9]. However, the need for expert knowledge in species-identification and survey methods is one of the greatest drawbacks of any multi-taxa approach [10]. Since habitat heterogeneity and complexity has long been recognised as an indicator of bird diversity [11–13], alternative biodiversity indicators use habitat features (such as patch density, canopy openness and forest area) as metrics, from which habitat health and biodiversity values can be inferred [14]. This kind of indicator can be extended to include the use of remote sensing methods. However, although remote-sensing methods can be used to predict potential areas of species-richness [13,15–17] and have been used to assess the effectiveness of certifications schemes [18], they cannot give actual biodiversity values [19]. The wealth of literature on selecting suitable indicators highlights a further obstacle to effective monitoring, whereby the global biodiversity dataset can be regarded as disaggregated, in part due to the non-standardised nature of data collection and lack of cohesion between different data-collecting groups [20,21]. Waldon et al. [22] suggest that a model protocol for biodiversity monitoring should be repeatable, robust against observer bias and require little training or equipment.

An acoustic community is defined as an aggregation of sound-producing species and as such can be considered an appropriate measure of biodiversity within a habitat [23]. The use of acoustic monitoring is emerging as a valuable tool in conservation. Acoustic surveys are considered to be a cost and time-efficient method for reliably sampling vocal communities [24]. However, processing recordings to identify species can be extremely time-consuming and susceptible to observer-bias. Research into automated species identification is growing but identifying species within variably noisy environmental recordings is problematic and not currently considered a suitable replacement for human processing [25]. Aside from these challenges, identifying species or groups of species is exclusive to other components of the soundscape which may be important for understanding the health of an ecosystem. The field of soundscape ecology is the study of the soundscape as a unit of measurement in and of its self [26]. It aims to understand the composition of sound energy in the context of the environment from which it emanates. The soundscape can be considered as the interaction between the biophony (sounds from animal sources); geophony (sounds such as wind and rain) and anthrophony/technophony (man-made noise pollution from machinery- typically between 1-2KHz) [26].

Within the biophony, there are three broad types of acoustic communities—infrasonic (eg. whales <20Hz); “ordinary” (20–20,000Hz eg.mostly within the human hearing range); and ultrasonic (eg. microchiropterans >20,000Hz) [23]. Within each of these acoustic communities there is further partitioning, which may be frequency or temporally bound. The niche hypothesis, first introduced by Bernie Krause [27], suggests that such frequency partitioning is a necessary evolutionary process that enables species to co-exist within acoustic space. Recent studies have shown that. the diversity and composition of a soundscape can be indicative of the richness of an acoustic community [28,29] and can offer insights into functional and phylogenetic diversity [30]. If used in conjunction with environmental data they may provide insights into habitat type [31] and of structural/vegetation characteristics within a habitat [32,33].

Aside from monitoring the biophony, the technophony is of key importance to biodiversity conservation. Noise pollution is known to have drastic impacts on faunal communities, including altering species communities [34]; masking acoustic signals in fish [35]; and causing birds to alter their song [36]. One study suggests that noise from machinery may alter the temporal dynamics and patterns of animal sounds within a soundscape indicating significant shifts in animal behaviours [37]. Perhaps one of the biggest contributors to the global technophony is road noise. The ecological effects of roads range from fairly obvious things such as increased mortality through traffic collisions (i.e. road kill) [38] to more obscure and sinister effects including increased habitat fragmentation and its associated effects [39]; and the accumulation of heavy metals and salts in roadside habitats, which can have consequences for terrestrial and aquatic wildlife [40]. Roads can also alter animal behaviours and road noise was shown to alter survival behaviours in the North American prairie dog *Cynomys ludovicianus* [41] and cause birds to change their calls to suit noisier city-habitats [36]. Understanding the dispersion of road noise through different habitats and how that contributes to the acoustic properties of a landscape is of key importance to furthering the field of soundscape ecology.

Several acoustic indices, which aim to quantify the soundscape, such as: the acoustic diversity index (ADI) and acoustic evenness index (AEI) [42]; the normalized difference soundscape index (NDSI) [43]; the acoustic complexity index (ACI) [29]; and the bioacoustic index (BAI) [44] have been developed in recent years. The use of these indices has been proposed as a low-cost, long term biodiversity monitoring strategy [45], which highlights the need to assess the suitability of these methods in a range of habitats. Plantation forests have become increasingly important refuges for biodiversity throughout the world [46]. There are 2.9 million hectares of woodland in Great Britain, and around 47% of it is coniferous plantations [47]. The majority of coniferous woodland is managed by clear-felling and replanting, thus creating mosaics of even-aged, mostly uniform, forest stands [48]. The resulting patchwork landscape offers opportunities to investigate species-assemblages within different aged stands and presents itself as a good model for studying soundscapes.

This is the first study to explore the relationship between the soundscape and physical properties of a coniferous plantation forest in the UK and it is structured around four objectives. Firstly, we explore how the soundscape changes with forest age, hypothesising that acoustic diversity would increase with age. Forests become more structurally complex with age [49], offering more resources and niches for bird species [11], which should in turn lead to a more complex acoustic community [45]. Secondly, we assessed the temporal variation of the soundscape (within and between years) in order to determine the potential suitability of these methods as a long-term monitoring tool. Thirdly as soundscape conservation is an important consideration both to ecosystem health and human health [50], we investigated how the acoustic signature of the forest changes with increasing distance from two moderately busy roads..

## Methods

Covering 18,730ha, Thetford Forest in East Anglia is the largest man-made forest in lowland UK [51]. Planted during the first half of the 20^th^ century on poor agricultural soils and heathland, it is a somewhat young forest, with forest stands ranging from 1 year to >100years, and is managed by the UK government Forestry Commission. Around 76% of the plantation comprises a mosaic of even-aged coniferous forest stands (of which 74% is Corsican Pine; 19% Scots Pine; and 7% other conifers), ranging in size from <1ha to 18ha, which are managed by clear felling and replanting on a 55–70 year rotation cycle. The remaining parts of Thetford forest comprise 12% broadleaf woodland (beech; oak; birch and mixed) and a mixture of open habitats including grassland, heathland, bracken and farmland habitats. The forest is divided by a number of roads, including three major ones (A1065; A134; A11) as well as a number of smaller ones. The forest is open to the public but vehicular access is restricted to Forestry Commission personal and those with permits. It is a popular recreational area and much of the forest is used by dog-walkers, cyclists, walkers and equestrians. There is a large deer population within the forest which is mostly managed by the Forestry Commission [52]. It is a designated Special Protection Area for the ground-nesting birds, the Woodlark and the Nightjar. It is also an important area for a number of scarce and rare fauna and flora in the UK and Europe and 94% of the forest (17,653ha) has been designated for national and international conservation interests [51].

**Fig 1:**
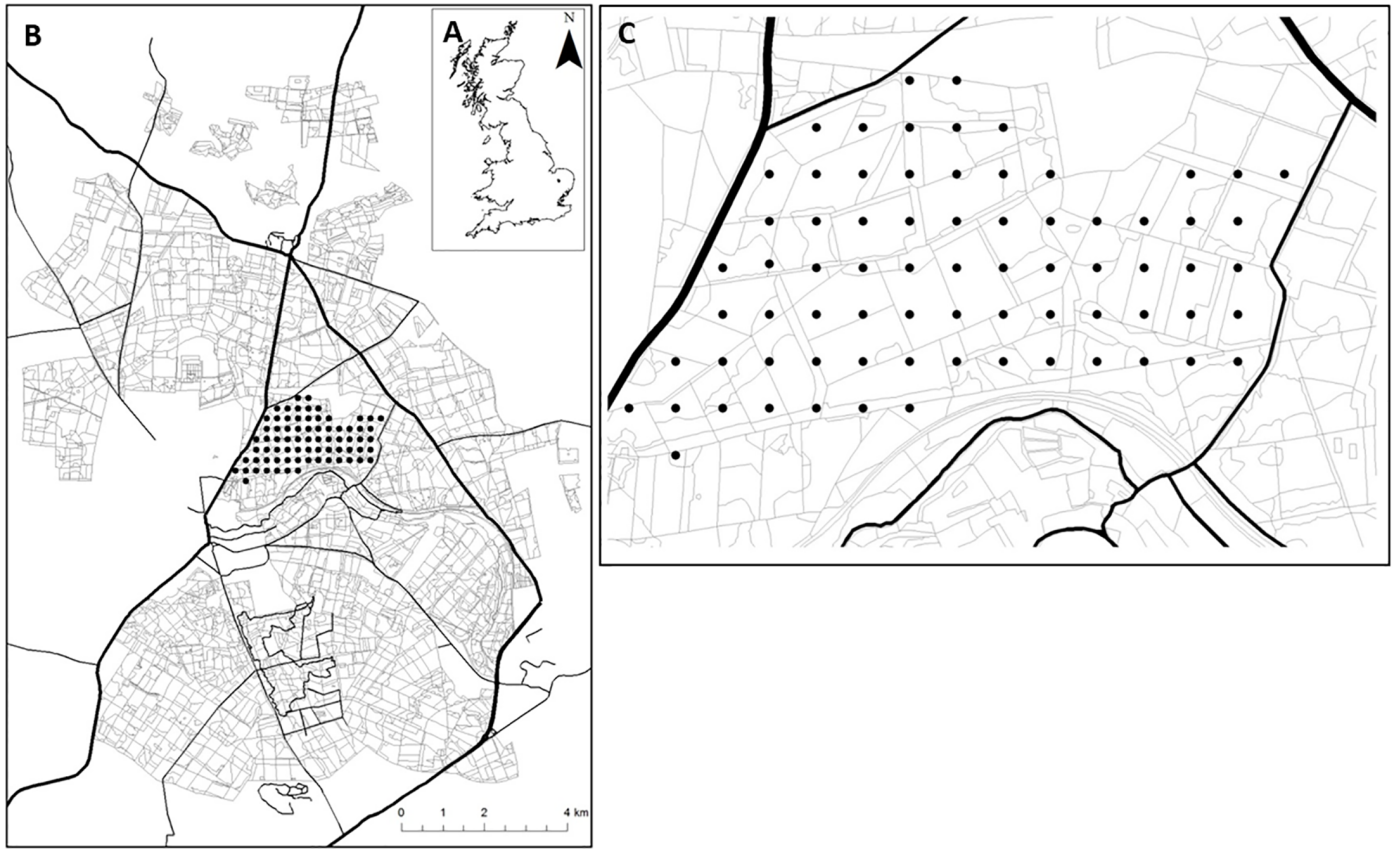
A) United Kingdom coastline. Thetford Forest (black dot) is situated in East Anglia. B) Map of the main central Thetford Forest block. Thick dark lines indicate busy A-roads. Thinner dark lines indicate minor roads. Black dots represent study grid. C) Study grid. Dots represent sampling points (n = 73), which are spaced 250m apart. Polygons represent different forest stands. The thick dark line on the Western edge of the grid is the A1065 and the thick dark line to the North East of the Grid is the A134 –two busy main roads.

This study took place near Santon Downham in the Central Thetford Forest block, which is managed by the Forestry Commission (52°28'2.1828''N, 0°39'53.2872''E) (Fig 1). Permission to conduct the study was obtained from the Forestry Commission and we liaised with them to ensure we did not interrupt any felling operations or deer management activities. Sampling points (N = 73) were predominantly classified as coniferous woodland (N = 65) of which 70% was Corsican Pine, 20% Scots Pine, 4% clear-felled and 6% other coniferous species; other habitats included broadleaved and lowland mixed deciduous woodland (n = 7) and grassland (n = 1).

### Recording methods and sampling design

To explore spatial variation in the forest soundscape, a systematic grid was used. Other soundscape studies have used sampling grids of 50m [33] and 100m [29]. In this study, a 250m grid was used (Fig 1), as one of the key objectives was to explore the relationship between forest stand age and acoustic diversity and a larger grid was necessary to represent a meaningful age-gradient of forest stands (Table 1). Soundscape recordings (44.1KHz; 24Bit; Stereo) were made using a Roland R-05 digital audio recorder and a DIY stereo microphone using Primo EM-172 electret condenser capsules, powered bya 5V phantom power battery box. Although the Primo EM172 capsules are omnidirectional, this stereo microphone was made to enable bi-directional recording for other purposes—a single Primo EM-172 capsule can be used to build a mono microphone and give suitable recordings for a study of this kind. The Primo EM-172 was selected as it is a favoured microphone for DIY nature sound recordists due to its low self-noise. Acoustic analysis of recordings was conducted on both channels and scores used in statistical tests were taken as the average value of both channels. The recording unit was mounted on a tripod 1.5m from the ground. To minimise handling noise, the observer stood 8m from the unit and remained quiet throughout recordings. Since the presence of an observer may affect the vocal activity of any animals, upon arriving at a site there was a one minute quiet interval before commencing recording. However, since Thetford Forest is a popular recreational area it was assumed that the observer did not have a drastic effect on the vocal fauna.

**Table 1:** Survey effort showing the age and type of forest stands sampled. CCA label column refers to the key for the Canonical Correspondence Analysis.

WOODLAND TYPE	AGE (YRS)	NO. SITES		CCA LABELS
		2014	2015	
Coniferous	0–5	7	8	CF1
Coniferous	5–10	7	7	CF2
Coniferous	15–20	9	9	CF3
Coniferous	20–30	6	6	CF4
Coniferous	30–35	9	9	CF5
Coniferous	35–45	13	11	CF6
Coniferous	45–50	8	10	CF7
Coniferous	50+	6	5	CF8
Broadleaf	15–20	1	1	BLM3
Broadleaf	50+	6	6	BLM8
*Lowland Acid Grassland (n = 1 both years)*				*LAG*

Three minute recordings were made at each grid point during the morning (6am-11am). Although this is a relatively short snapshot of a soundscape, it is possible to capture and categorise different habitats using as little as 80 seconds of recording [31]. Furthermore, our aims were to investigate the role that snapshot soundscape recordings might have in large-scale monitoring so shorter recordings and more sites were deemed a suitable logistical trade-off. On average, it was possible to visit 21 sites during the 6-11am sampling window. To ensure each site was sampled earlier and later during this period, it was further split into two separate sampling periods (6am-08:30am and 08:30am-11am). The temporal variation of the soundscape between different years was explored by sampling the grid in two consecutive years during the summertime: between 21^st^ July–20^th^ August 2014; and 29^th^ July–28^th^ August 2015. Due to logistical limitations it was not possible to sample all sites equally but most sites were sampled at least six times in each year, with each site being sampled at least three times in one or both sampling periods. During 2014, two sites were sampled five times, 61 sites sampled six times, and ten sites were sampled seven times (N = 446). In 2015 three sites were sampled five times, 64 sites sampled six times, four sites sampled seven times and two sites were sampled eight times (N = 443).

Percentage cloud cover was recorded for each recording by estimating what portion of the sky directly above the observers head and in the observers field of view was taken up with cloud (0–20%; 20–40%; 40–60%; 60–80%; 80–100%). This was done from the closest track from each recording point since in some sites it was not possible to see a representative portion of the sky. Recordings were not made if it was raining or in very windy conditions. Due to logistical constraints an anemometer was not available for the study but the wind levels were estimated for each recording as 1 = still; 2 = light breeze; 3 = strong breeze; 4 = windy. However, for analysis purposes this was condensed to whether it was still or if there was any breeze (1 = no wind; 2 = some wind). The final “wind” score for each site was calculated as the percentage of “windy” recordings. To assess the suitability of this, we also obtained hourly wind-speed records from the nearest UK Met-Office weather station at RAF Marham (14miles north of study site). Although this is quite a distance, we make the assumption that the weather will have been similar at our study grid, largely due to the (flat) topography of East Anglia.

### Vegetation structure and landscape variables

Field-based observations and GIS data were used to identify habitat structure and landscape characteristics. Canopy height was estimated using an ordinal scale of: 0m; 1-5m; 5-10m; 10-15m; 15-20m; 20-25m; >25m. Canopy cover was estimated using an ordinal scale of: 0% cover; 1–20%; 20–40%; 40–60%; 60–80%; 80–100%. Tree density was measured by counting the number of trees above head height (ca. 2m) in an 8m radius of the recording position. The number of different tree species (referred to as TRSP from here on) and types of ground vegetation were noted down for the same 8m radius. Average ground vegetation height was estimated using an ordinal scale of: 0; 0-10cm; 10-40cm 40-80cm; 80-130cm; >130cm). Vegetation surveys were conducted in both years. GIS data obtained from the Forestry Commission was used to determine the age of each forest stand on the grid and the number of species planted in each stand (from here on referred to as ‘stand diversity’). The distance of recording points to the nearest road (50m bands) and the nearest forest stand edge (10m bands) were measured using the multiple ring buffer and intersect functions in ArcGIS.

### Calculation of acoustic indices

In order to improve processing time in R, each three minute recording was split into ten second segments using WAV Splitter v.1.31 (DigitByte Studio software). Unwanted noises (i.e. footsteps, handling noise) were removed from subsequent analyses by removing relevant segments (generally 30s from the beginning and 30s from the end). For each recording, acoustic indices values were calculated for 12, ten second segments (equating to two minutes in total). The average value of these 12 segments was then used for that particular recording. Recordings from both sampling periods (early morning and late morning) were pooled. In the site comparison analyses, the average acoustic indices values were used. (ca. 12 minutes audio per site). There were a total of 453 soundscape recordings from 2014 and 445 soundscape recordings from 2015. This equates to 889 two minute samples (2014 N = 446; 2015 N = 443). As Thetford Forest is a recreational area, popular with dog-walkers and cyclists, some recordings were interrupted when the observer was approached by other forest users. In these instances, if the full three minute recording had not been collected the observer returned to that point within 15 minutes and collected the remaining recording period required. The usable parts of the two recordings were considered as one repetition for analysis purposes. A total of 29.6 hours of recordings (2014 = 14.9hours; 2015 = 14.7hours) from 73 locations were considered in the analyses.

Five acoustic indices were calculated for each soundscape recording using the *soundecology* package [53] in the R (ver3.1.3) statistical analysis environment [54]. The acoustic diversity index (ADI) and acoustic evenness index (AEI) were calculated using values derived from the proportion of sounds above -50dbfs (decibels) in ten 1 KHz frequency bands across the 0–10 KHz frequency range [42]. The normalized difference soundscape index (NDSI), which calculates the ratio between anthrophony and biophony [43], was calculated using default bandwidth values (i.e. anthrophony = 1-2KHz; biophony = 2-11KHz). The acoustic complexity index (ACI) was developed to sample avian communites and essentially divides the recording into frequency bins (i.e. bands) and temporal steps and then calculates the sound intensities within this matrix, giving a measure of the number of sound events and their relationship to one another [29]. In this study it was calculated using the default parameters (Fast Fourier Transform window length = 512; cluster size (J) = 5). The bioacoustic index (BAI) measures the amount of sound intensity (y axis) across a specified frequency range (x axis) and the index value is essentially the area under the curve for any given recording. It was calculated for sounds between 2–8 KHz and thus serves as a function of the sound levels and frequency bands used by the majority of avifauna [44].

### Data analysis

To investigate the relationship between the soundscape and environmental variables in coniferous forest stands only (N = 65) a Spearman Rank correlation matrix was created using SPSS v.23 [55]. This matrix was used to identify and interpret the nature of the relationships between indices and environmental variables and to guide subsequent analyses.

We tested our variables for normality using Shapiro-Wilk tests and Q-Q plots and applied square-root transformations to achieve normality where appropriate (indicated with sqrt in Results section). In cases where transformations did not achieve normality, we used non-parametric equivalent tests. To address the hypothesis that acoustic diversity would increase with stand-age we grouped forest stands based on age and conducted one-way ANOVAs with Gabriel post-hoc tests using SPSS v.23. Gabriel post-hoc tests are recommended where samples sizes are unequal [56]. Homogeneity of variance was tested using the Levene test. The Kruskall-Wallis test was performed on ACI values from 2014 and 2015 as these data failed the Levene test.

Canonical correspondence analysis [57], using PC-Ord v.6 (MjM Software, Oregon, USA), explored the correlation between sounds occurring in different frequency bands with habitat variables and stand age. The frequency band values used represent the proportion of sounds above -50dbfs (decibels) in ten 1 KHz frequency bands (0-10KHz). CCA is often used in ecological studies to investigate the associations of different species with habitat features and types. Here, we enter the frequency band values as we would with species count data, (N = 10) since we were interested in determining where these frequencies lie within the physical landscape. Because species’ calls span different frequencies, ordinations can be used to determine which frequencies are most associated with particular habitat features and shed light on the acoustic community present [32,33]. All sites (N = 73) were included in the CCA and were split into 11 categories based on stand-age and habitat-type (Table 1). Habitat structure metrics were averaged for each category and each category classed as a site. To investigate how the anthrophony/biophony ratio (i.e. NDSI) changes with increasing distance from roads, linear regression was used. The impact of road noise on the landscape was visualised using interpolation maps created in ArcGIS [58].

To assess whether acoustic indices values from each site (N = 73) were similar between years, parametric (i.e. Pearson) (ADI, AEI, NDSI, BAI) and non-parametric (i.e. Spearman–rank) correlations (ACI) were used. To determine whether indices values per site were significantly different between years, we used paired-samples t-tests (ADI, AEI, NDSI, BAI) and Wilcoxon signed-rank tests (ACI). To investigate correlations between weather data (personal observations and weather station data) and acoustic indices scores we used a spearman rank correlation matrix. To investigate differences in the soundscape between the two recording periods (i.e period one = 06:00–08:30am and period two = 08:30–11:00am), paired samples t-tests were performed where appropriate and a Wilcoxon Signed-rank test was used for non-normally distributed data.

## Results

### Relationships between the soundscape and environmental variables

Spearman correlations between acoustic indices and environmental data in coniferous forest stands (Table 2) suggest that ADI was higher in older forest stands with a taller, more closed canopy. These stands were more likely to have a higher diversity of tree species and lower ground cover diversity. Inversely, AEI was lower in these taller, more closed stands. ACI was higher in stands with a more open canopy/no canopy and higher ground cover diversity. These stands tended to be younger, with a shorter canopy and lower tree density. ACI was the only index to display a relationship with distance to the forest stand edge, indicating that ACI tended to be higher in more open areas. NDSI increased with increasing distance from the nearest road in both years. In 2014, NDSI also bore a relationship with stand age though this relationship was not observed in 2015. In 2014 BAI bore a relationship with canopy height and had a similar strength relationship to stand age as ACI displayed. However, in 2015 these relationships were not observed.

**Table 2:** Spearman rho correlation matrix of acoustic indices and environmental variables for coniferous woodland (N = 65).

		Age	RdDist	EdgeDist	STDV	TRDN	TRSp	CNHT	CCVR	GCDV	GCHT
**2014**	ADI	.570**	.038	.151	.225	.034	.485**	.570**	.503**	-.014	-.087
	AEI	-.559**	-.080	-.109	-.206	-.039	-.470**	-.555**	-.482**	-.034	.096
	NDSI	.374**	.659**	-.239	.109	-.177	.275*	.151	.150	.243	.102
	ACI	-.274*	.108	-.367**	-.047	-.309*	-.128	-.344**	-.536**	.282*	.291*
	BAI	.321**	-.111	.077	.010	-.078	.215	.421**	.264*	.047	.153
**2015**	ADI	.659**	.283*	.121	.446**	.233	.346**	.646**	.665**	-.307*	-.084
	AEI	-.643**	-.309*	-.082	-.463**	-.215	-.373**	-.656**	-.601**	.261*	.034
	NDSI	.213	.640**	-.212	.210	-.141	.207	.248*	.041	.033	-.066
	ACI	-.321**	.046	-.320**	-.023	-.430**	.148	-.237	-.525**	.376**	.021
	BAI	-.005	-.199	-.063	.027	-.047	.125	.089	.122	-.068	-.046

Non-significant results are displayed as ns.

Significant correlations are marked in bold:

** = p<0.01;

* = p<0.05).

GIS data: Age = forest stand age; RdDst = distance to nearest road; EdgeDST = distance to edge of forest stand; STDV = stand diversity—no. species planted by the Forestry Commission. Field data: TRDN = tree density; TRSP = no. of different tree species; CNHT = canopy height; CCVR = canopy cover; GCDV = number of different types of ground vegetation; GCHT = ground vegetation height

### Stand age

**Fig 2:**
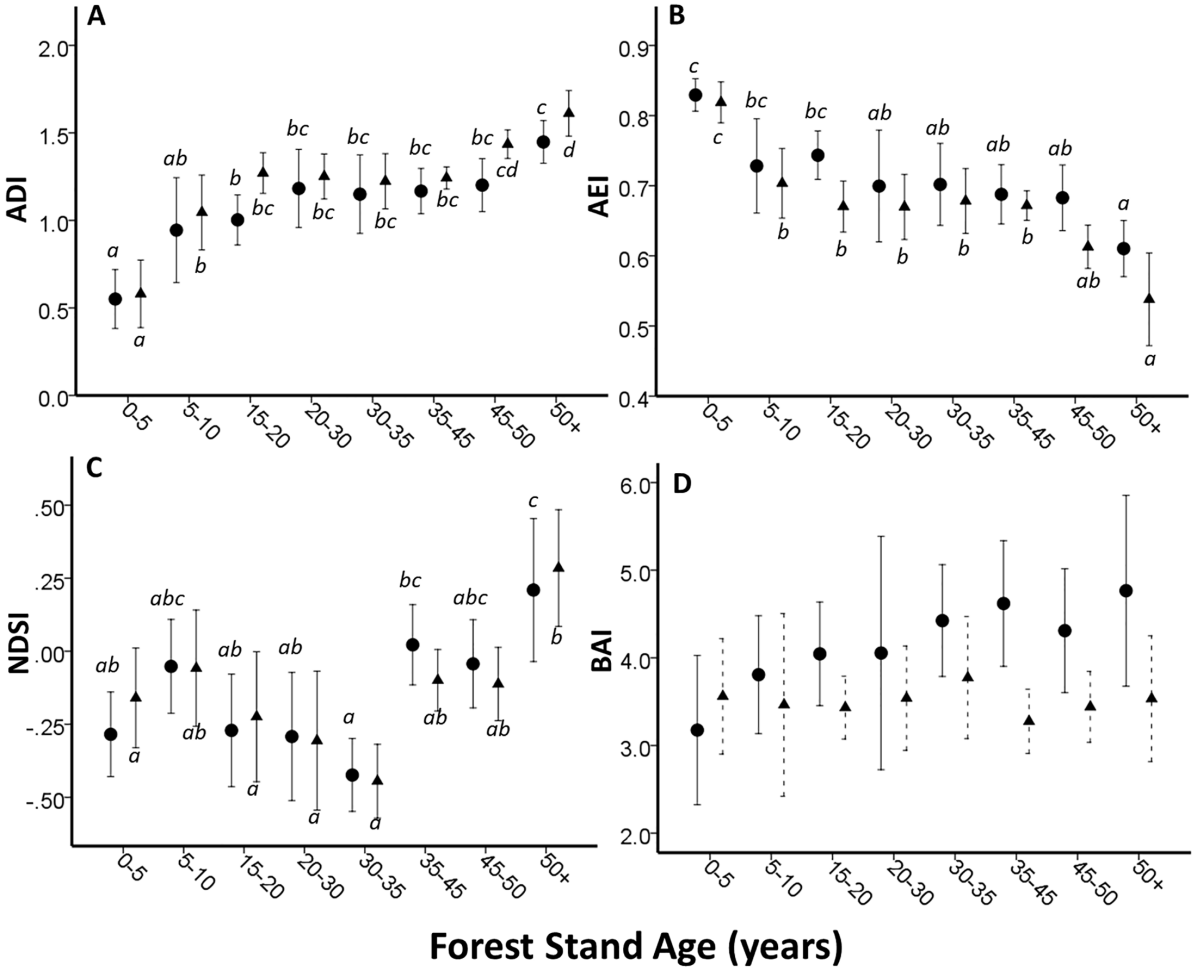
Relationships between acoustic indices with coniferous forest stand age (N = 65) from each year (dots = 2014 data; triangles = 2015 data). Letters represent mean groupings from the Gabriel post-hoc test (p<0.05). A) Mean ADI (+/-2 SE). 2014, F_7,57_ = 6.896, p<0.001; 2015, F_7,57_ = 18.772, p<0.001. B) Mean AEI (+/-2 SE) 2014, F_7,57_ = 5.417, p<0.001; 2015, F_7,57_ = 14.359, p<0.001. C) Mean NDSI (+/-2 SE) 2014, F_7,57_ = 5.827, p<0.001 (*abc*); 2015: F_7,57_ = 5.010, p<0.001. D) Mean BAI—there were no significant differences between age-groups but the plotted means indicate that in 2014 the mean BAI values were higher in older stands.

One-way ANOVAs reveal significant differences in ADI/AEI and NDSI in different aged forest stands (Fig 2). ADI was higher in older forest stands and lowest in the youngest, more open stands. There appears to be a non-linear relationship, which shows two stages of increase in acoustic diversity, which is corroborated with Gabriel Post Hoc test—showing three (2014 data) and four (2015) groupings of sites based on mean values (Fig 2). AEI displays the inverse relationship to that of ADI. Groupings based on NDSI values are less clear, which is likely due to the effect road noise at different sites. Since NDSI calculates the ratio between biophony and technophony, the weaker observed relationships with stand age were somewhat expected. ACI was significantly different in different aged forest-stands (Kruskal-Wallis; 2014, *X*^*2*^ = 20.932; p<0.005; 2015 *X*^*2*^ = 20.327; p = 0.005) and visual inspection of the data indicate that this is largely driven by high ACI values in the youngest forest stands. There were no significant relationships observed between BAI and different age-groups of forest. However, plotting the mean values from 2014 revealed a similar relationship to that observed between ADI and stand age but this relationship was not as clear in 2015 (Fig 2).

### Canonical correspondence analysis

**Fig 3:**
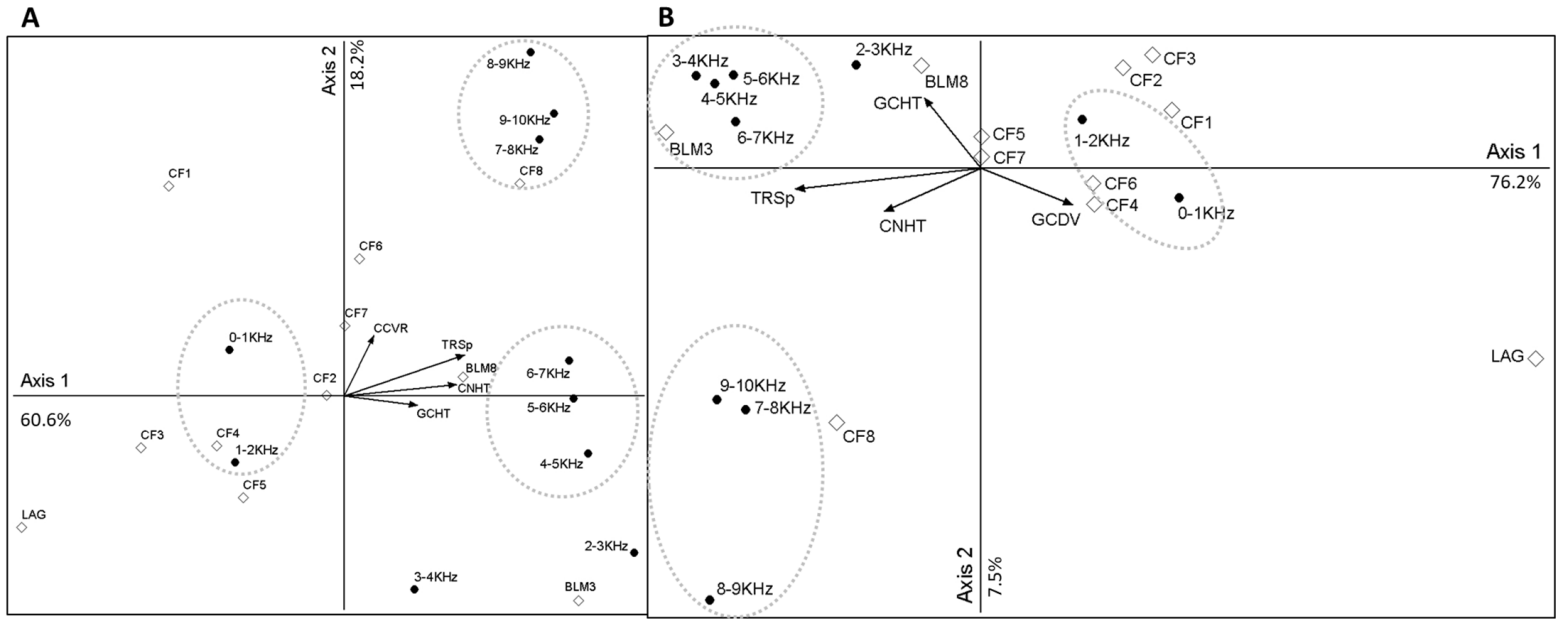
Canonical correspondence analysis exploring the relationship between habitat type habitat features and ten 1KHz frequency bands. (see Table 1 for key to category labels; R^2^ cut-offs for environmental variables = 0.1; TRSp = no. of tree species; CNHT = Canopy Height; GCHT = ground vegetation height; GCDV = ground vegetation diversity). A) 2014 data. Strong associations between axis 1 with CNHT and TRSp (S1 Table) indicate that as structural complexity increases, the higher frequency bands become more apparent in the soundscape. B) 2015 data. Similar relationships between axis 1 and habitat structural metrics (S1 Table) show fairly similar distribution of sites in relation to frequency bands. See Results section for explanation of key findings.

The ordinations reveal that there were three approximate frequency band clusters: 0-2KHz (associated with more open habitat types), 4-7KHz (associated with broadleaf woodland), and 7-10KHz (older coniferous woodland) (Fig 3). They also suggest that acoustic diversity would increases with habitat structural complexity. Axis 1 in both ordinations was strongly associated with the biophony (3-10KHz), which was higher in stands with greater structural diversity. Axis 2 reveals that older coniferous sites (>35 years) were most associated with the highest frequency bands (7-10KHz) in 2014, but only the oldest group (>50 years) was associated with this bandwidth in 2015. Open areas were also associated with these high frequency bands (CF1 in 2014 and LAG in 2015). Across all axes, sites aged 15-35years (CF4, CF5, CF6) were largely similar to one another in their regression scores, which may partly explain the ‘plateau’ in acoustic diversity revealed in earlier analyses.

### Anthropogenic disturbance

**Fig 4:**
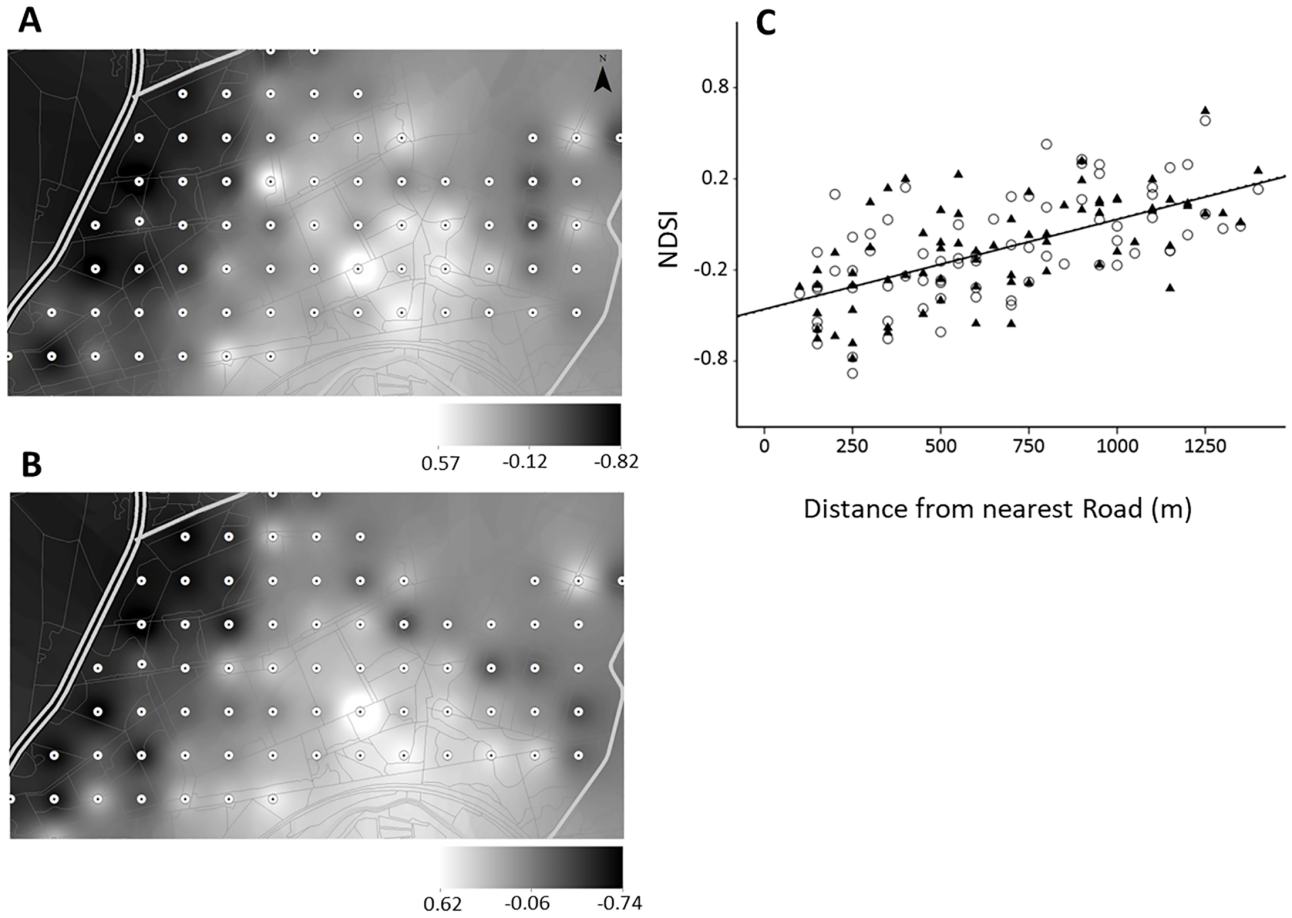
Interpolation maps of average normalised difference soundscape index (NDSI) scores from each sampling point from 2014 (A) and 2015 (B) (points are 250m apart). Darker shading indicates higher levels of anthropogenic/technophonic sounds (i.e. from machinery) in the soundscape. Lighter shades indicate higher levels of biological/biophonic sounds. The grey and black striped line on the left of each map is the A1065, a busy main road. The lighter grey lines indicate smaller connecting roads. The lighter grey lines indicate smaller connecting roads. C) Strong positive relationship between NDSI and distance to nearest road 2014 data (r2 = 0.373, p<0.001, N = 73) displayed as circles; 2015 data (r2 = 0.397, p<0.001) displayed as black triangles. The lines of best fit for both datasets overlap so are not distinguishable from one another.

The strongest predictor of NDSI in both years was the distance to the nearest road (Fig 4). The sampling grid was sandwiched between two busy roads, the A1065 (running along the Eastern edge of the grid) and the A134 (ca. 500m West of the grid). NDSI values reached ‘0’ (i.e. an equal amount of anthrophony/biophony in the soundscape) at approximately 1km from the nearest road. ADI and AEI were also significantly correlated with distance to nearest road but the strength of the relationship is somewhat lower. This is likely due to the way the indices are calculated (see Methods section), making NDSI a more suitable measure of road noise/anthropogenic disturbance.

### Temporal changes in the soundscape

Pearson correlations reveal that ADI, AEI and NDSI values from 2014 were strongly related to 2015 values for ADI r = 0.709; AEI r = 0.646; and NDSI r = 0.810, but only displayed a moderately weak correlation for BAI(sqrt) r = 0.381, p<0.001, N = 72 for all correlations). ACI values from 2014 were related to 2015 values (Spearman Rho r_s_ = 0.428, p<0.001, N = 72). Paired-samples t-tests reveal that mean ADI per site was higher in 2015 (t_71_ = 4.78 p<0.01), whilst AEI was lower (t_71_ = -4.93, p<0.01). BAI (sqrt) was significantly higher in 2014 than 2015 (t_71_ = 4.742 p<0.01). Neither NDSI or ACI were significantly different between years.

The number of “windy” recordings was considerably higher in 2014 (N = 264) than 2015 (N = 165). The proportion of windy recordings (from here referred to as %WND) was significantly correlated to mean windspeed (kn) data obtained from the met-office (Spearman Rho, 2014 r_s_ = 0.279, p<0.05; 2015 r_s_ = 0.48, p<0.001), indicating that our rough measures of “wind” reflected that of the observed windspeeds at RAF Marham. Relationships between %WND and acoustic indices were stronger in 2015 than 2014. ADI was lower when %WND was high (2014, Spearman Rho r_s_ = -0.247, p<0.05; 2015, r_s_ = -0.475 p<0.001) and in 2015 AEI was higher when %WND was high (r_s_ = 0.427 p<0.001). Similarly ACI bore a significant correlation to %WND in 2015 only (Spearman Rho r_s_ = 0.493, p<0.001), indicating that ACI is higher during “windy” recordings.

Acoustic activity was significantly higher during the first recording period (06:00–08:30) (ADI 2014, t_72_ = 5.233, p<0.001; 2015, t_72_ = 2.025, p < 0.05; and BAI 2014, t_72_ = 4.874 p<0.001; 2015, t_72_ = 2.850, p<0.01). AEI and NDSI were higher during the second period (08:30–11:00) in 2014 (t_72_ = -2.929, p<0.005 and t_72_ = -2.076, p<0.05 respectively). NDSI was also higher during the second period in 2015 (t_72_ = -2.001, p<0.05), which may be related to an easing of traffic after morning “rush hour”. ACI was not significantly different between different recording periods.

## Discussion

### Relationships between the soundscape and environmental variables

The relationships between ADI and AEI with canopy characteristics and forest stand age echo the findings of Pekin et al. [32], who observed a similar relationship in a Costa Rican rainforest. The relationship in this study perhaps reflects the management strategy of the Forestry Commission. Trees in the newly established stands were planted in rows ca. 2m apart, with trees spaced ca. 1m from one another. Stands aged between 10–35 years were typically very dense due to natural establishment of new trees, and often had little or no ground vegetation cover. From around 20 years old, stands are progressively thinned every five years until the remaining timber reaches economic maturity between 55–70 years [51]. Thinning opens up the forest and allows light to reach the forest floor, enabling a more complex ground vegetation to establish itself [59].

**Fig 5:**
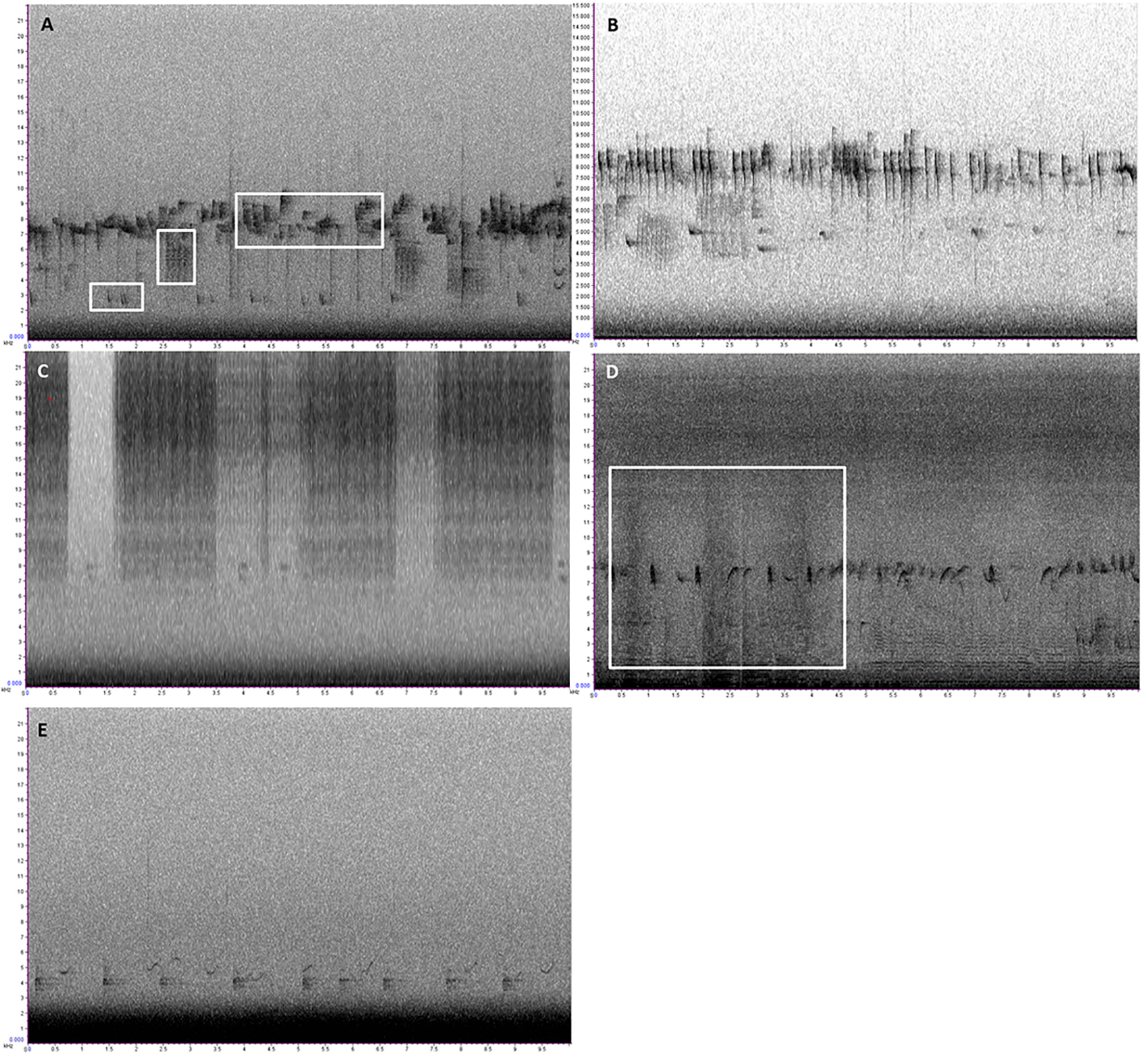
Ten second spectrograms illustrating different contributors to the soundscape. A) An example of a recording with high ADI, BAI and NDSI values. Three separate bird species calls are highlighted by white squares. This kind of frequency partitioning is one of the key concepts in soundscape ecology and ecoacoustics. B) Recording from an older coniferous stand (>45yrs) capturing what appear to be contact calls of *Regulus regulus* (goldcrest). C) Highlighting the presence of Orthoptera within our recordings. Although we only utilised audio data up to 11KHz in our statistical analyses, this recording shows that the Orthopterans in our recordings are occupying higher frequencies (up to 22KHz). D) Another recording with high values in the 0-10KHz range. The three darker vertical patches highlight the sound of a flying insect (potentially Syrphidae) passing the microphone. This kind of frequency modulated pattern might be a useful indicator of winged-insect (i.e. pollinator) activity. E) Recording displaying very low NDSI values (-0.95 –i.e. high road noise) which is evident from the thick black band filling the 0-2KHz frequency range.

It has long been understood that as habitat structural complexity increases, so too does bird diversity [11]. Mean number of individuals (birds) increases linearly with woodland age for winter-bird communities in UK plantation forests but community composition is more dependent upon structural characteristics [60]. Calladine et al. [61] found that bird assemblages in young UK coniferous forest stands (<10years) were typically distinct from older stands (15-30years). Although we have no biodiversity values for our study grid, our results indicate that different aged stands comprise different compositions of sound energy and older stands generally have higher levels of acoustic diversity. This may be caused by higher levels of bird vocal activity (i.e. one bird/species with a large vocal repertoire) or higher levels of bird diversity (i.e. more vocal species). Upon further investigation, the ordinations suggest that as structural complexity increases, the higher frequency bands (4-10KHz) become more represented in the soundscape—the majority of UK woodland bird calls range between 3-8KHz. Visual inspection of spectrograms highlight the contributions of different bird calls to the soundscape (Fig 5). In the oldest coniferous stands (>50years), the highest bandwidths (7-10KHz) become particularly noticeable within the soundscape. The goldcrest (*Regulus regulus*), which has one of the highest frequency calls in the UK (peak frequency ca. 7KHz, with contact calls peaking higher still) is more associated with older coniferous woodland than other habitats in the UK [60] and so may have been a key contributor to the soundscapes of the oldest coniferous stands in this study (Fig 5).

Another contributor to the high frequency bandwidths is likely to be the wing beats of Dipterans, in particular the Syrphids (hoverflies), and flying Hymenopterans (bees and wasps). Syrphid diversity is generally greater where there is greater ground vegetation cover [62]. Hoverflies were present in many of the older forest stands during the study, and the ambient background noise in some stands was a gentle buzzing sound (pers. observation). This buzzing sound can increase the overall proportion of sound within a recording and when a flying insect passes close to the microphone the buzzing sound can occupy the entire bandwidth of the recording (0KHz-22.5KHz) and is strong at 10KHz (Fig 5). In areas with greater ground vegetation complexity and flower diversity, there are likely to be a higher number of flying invertebrates which may explain why some of the more open areas had similar sound profiles to the older forest stands with regards to the higher frequency bands (8-10KHz). Orthopterans (crickets and grasshoppers) were also more abundant in the open areas (pers. observation) and their songs can occupy a wide range of frequencies, including 10KHz [63] (Fig 5). The grassland site (LAG) was more strongly associated with the higher frequency bands in 2015 than in 2014, which may be due to the grass being cut by the Forestry Commission during the 2014 field season and subsequently disturbing the invertebrate communities there. Mowing events cause major Orthopteran population declines [64] with diversity and abundance recovering over time [65]. However, due to the relatively short period of time we were sampling, it is unlikely that the Orthopteran communities re-established themselves during the remaining sampling period. Our findings with regards to ACI were in contrast to those found in a study by Farina and Pieretti [33], who found that ACI was typically higher in sites with denser vegetation. This study observed the opposite pattern, where ACI was generally higher in more open areas. This may be explained by the strong effect that the wind had on ACI. However, it may also have been partly driven by the presence of invertebrate communities in the more open areas since ACI was calculated for the whole bandwidth of the recordings. The lack of strong relationships between BAI and any habitat characteristics could perhaps be explained by the relatively broad way in which BAI characterises the soundscape. It was developed to detect differences in bird communities in rich bird-diverse rainforests on Hawaii [44]. This may explain strong differences in BAI between recordings made during the dawn chorus and those made shortly afterwards. But perhaps due to Thetford Forest being relatively species-poor poor when compared to Hawaiian rainforests, BAI was not acute enough to detect changes in the soundscape along the age-gradient.

### Anthropogenic disturbance

Anthropogenic noise disturbance is recognized as a threat to terrestrial wildlife [66]. At high levels it can cause birds [67] and frogs [68] to alter their song characteristics to reduce signal masking. Bats will avoid crossing roads where vehicle noise reaches a certain level [69]. Mcclure et al. [70] created a phantom road (using loud speakers in an otherwise road-free area) and observed that bird abundance declined by over 25% during periods of road-noise and that two species completely avoided the phantom road. The negative impacts that anthropogenic noise can have on humans is also well documented, with a whole quite of [71]. Indeed the practise of forest bathing (or *Shinrinyoku* in Japanese) is a means of escaping the stresses of city-living and can have measurable health benefits to humans [72], part of which could be due to being immersed in a natural soundscape. Natural sounds have been shown to speed up recovery from stressful situations in humans [73], and to reduce stress-levels in coma patients [74].

Recognising the negative impacts that anthropogenic noise has, and the positive benefits that natural soundscapes can have, on biodiversity and on human health, Dumyahn and Pijanowski [75]suggest that soundscapes should be viewed as a common pool-resource and be managed as such. Other studies have documented ‘user’ perceptions of soundscapes in national parks [76] and urban green space [77] to identify management and mitigation needs. Using the NDSI, this study demonstrates how noise from a busy road leaches into the forest (Figs 4 and 5), with a balance between anthrophony and biophony (i.e. NDSI = 0) being reached at ca. 1km from the nearest busy road. This distance echoes findings from a 2010 meta-analysis of road-impact studies which found that bird communities were affected over a distance of up to 1km [78]. This distance may differ depending on the size of the road and how busy it is, the type of surrounding habitat and time of day. However, we demonstrate the potential for NDSI to be used as a tool for modelling and predicting areas of ‘acoustic tranquillity’, and for managing soundscapes and mitigating noise disturbance.

### Temporal changes in the soundscape

Interpolation maps help to visualise the relationship between both sampling years in terms of the acoustic signature (S1 Fig). The soundscape on the grid did change between years but the general patterns of acoustic diversity remained largely similar with regards to stand structure. Changes in bird assemblage structure due to migration and/or breeding season success may account for some of these differences. Since bird communities were not sampled as part of this study, it is not possible to determine whether a change in bird communities was the main driver of the observed differences. However, the ordination techniques do offer some evidence that differences in ADI/AEI were potentially being driven by changes in bird communities. Furthermore, changes in ADI/AEI over the five hour sampling period also indicate that these indices were detecting changes in bird activity. ADI was higher between 6-8am, which corresponds with the ‘dawn chorus’ peak of bird activity.

Perhaps a more likely explanation for the observed differences in ADI/AEI between sampling years was the differences in wind conditions in each year. It is clear that the wind affects the performance of some acoustic indices as proxies for species-richness [79]. All of the acoustic indices used in this study were strongly affected by the wind conditions. With the proliferation of studies using automated recording units (ARUs), understanding the relationship between the geophysical properties of a soundscape and the performance of acoustic indices will become even more important for ensuring accurate predictions about biodiversity can be made using unattended field recordings. Deploying low-cost weather stations alongside ARUs in the field may help to disentangle the geophony from the biophony and anthrophony. They may also prove useful for understanding the geophonic properties of different habitat-types, since the wind-profile of a forested landscape is shaped by canopy structure and tree density [80]. In the very least, they would help to speed up the processing of large numbers of recordings by allowing researchers to rule out windy/rainy/stormy recordings more efficiently.

Our study was conducted by one observer using one recording unit on a minimal budget. This is important to note as the FSC principles and criteria state that scheme participants should conduct biodiversity monitoring that is relevant to the scale of their operation [81]. This presents a challenge as the monetary resources and expertise of those collecting data will vary greatly. The observer bias of using a soundscape approach would mostly rest on the type of recording equipment used. This bias could be minimised by using the same equipment; or by calibrating different types of equipment to enable comparisons of data collected with different units. Farina *et al*. [82] demonstrated that low cost recording units detected similar patterns in acoustic complexity to more expensive recording equipment, though overall resolution was reduced. The use of automated recording units (ARUs) is becoming more common in the field of soundscape ecology. However, ARUs are relatively expensive and typically do not provide data on weather conditions, although some units are now available with on board sensors for light, humidity, temperature and pressure [83], which can greatly affect the interpretation of data. Using handheld recording units and a more traditional on-foot approach may be more suitable for wider monitoring applications.

## Conclusion

The relative low-cost of the recording equipment used in this study would enable regular assessment of forests to inform adaptive management strategies. The need for a relatively high-powered computer is the main logistical barrier to this kind of monitoring being used for certification schemes or other environmental initiatives. However, having a centralised data processing location as part of a monitoring initiative may reduce the monetary pressure on scheme participants (such as in the FSC) and enable for better data management protocols to be put in place [84]. Our results indicate that older forest stands have higher acoustic diversity, which could be explained by changes in the vocal community. This relationship could be used to measure the progress and impacts of management decisions on biodiversity. The suite of acoustic indices currently available offers a number of ways to characterise the acoustic landscape. For example, the NDSI could be used to produce dispersion models of road-noise. Used in conjunction with species-distribution data such information could feed into landscape planning and noise mitigation strategies. Interpolation maps of both NDSI (Fig 4) and ADI (S1 Fig) demonstrate how such soundscapes can be visualised to aide interpretation and highlight areas of interest. Methods from soundscape ecology clearly have great potential for the conservation and management of forests and biodiversity. Collaborations between soundscape ecologists and species-focussed research will likely add another string to the bow of our understanding of how anthropogenic activities impact on nature.

## Supporting information

S1 Table Correlation matrix showing intra-set correlations between environmental variable and three ordination axes of the CCA. **Bold** values show positive relationships.
(DOCX)

S1 Fig Interpolation maps of ADI values across the study grid. A) 2014 data. B) 2015 data. Darker areas indicate lower acoustic diversity. Site labels are those used in CCA analysis and a key can be found in Table 1 in main body of text. These maps highlight how the acoustic diversity of the area displayed a similar pattern in both years. The dotted circular line (in both maps) shows site 63, which was felled between sampling years and so displayed major changes in the soundscape. These maps highlight the potential for using such sound-mapping techniques for monitoring change between years. Further research into selecting appropriate resolutions in different habitats is key to optimising performance of such tools.
(TIF)
