# Supplementary material for: Sound-mapping a coniferous forest—Perspectives for biodiversity monitoring and noise mitigation
Source: PLoS One. 2018 Jan 10;13(1):e0189843. doi: 10.1371/journal.pone.0189843 (PMC5761852; doi:10.1371/journal.pone.0189843)
Supplement: S1 Table — Bold values show positive relationships. (DOCX) [file pone.0189843.s001.docx]

| Variable | Axes 2014 | | | Axes 2015 | | |
| --- | --- | --- | --- | --- | --- | --- |
|  | 1 | 2 | 3 | 1 | 2 | 3 |
| TRDN | -0.303 | **0.177** | **0.544** | -0.028 | **0.016** | **0.916** |
| TRSp | **0.805** | **0.364** | **0.375** | -0.932 | -0.184 | -0.122 |
| CNHT | **0.749** | **0.099** | **0.001** | -0.486 | -0.386 | **0.36** |
| CCVR | **0.2** | **0.541** | **0.469** | -0.37 | -0.275 | **0.594** |
| GCHT | **0.489** | -0.087 | -0.681 | -0.284 | **0.631** | -0.404 |
| GCDV | **0.407** | -0.207 | -0.846 | **0.462** | -0.329 | -0.647 |
| 0-1KHz | -0.325 | **0.133** | -0.146 | **0.511** | -0.076 | -0.165 |
| 1-2KHz | -0.309 | -0.190 | **0.185** | **0.260** | **0.125** | **0.277** |
| 2-3KHz | **0.820** | -0.448 | -0.100 | -0.322 | **0.266** | **0.419** |
| 3-4KHz | 0.199 | -0.555 | **0.310** | -0.734 | **0.239** | -0.142 |
| 4-5KHz | **0.690** | -0.165 | -0.209 | -0.687 | **0.219** | -0.276 |
| 5-6KHz | **0.649** | -0.008 | -0.292 | -0.638 | **0.241** | -0.250 |
| 6-7KHz | **0.636** | **0.101** | -0.144 | -0.633 | **0.121** | -0.195 |
| 7-8KHz | **0.552** | **0.736** | **0.218** | -0.607 | -0.622 | **0.196** |
| 8-9KHz | **0.528** | **0.986** | **0.597** | -0.699 | -1.111 | **0.275** |
| 9-10KHz | **0.593** | **0.809** | **0.527** | -0.681 | -0.596 | -0.498 |
