# Supplementary figures and images for: Sound-mapping a coniferous forest—Perspectives for biodiversity monitoring and noise mitigation
Source: PLoS One. 2018 Jan 10;13(1):e0189843. doi: 10.1371/journal.pone.0189843 (PMC5761852; doi:10.1371/journal.pone.0189843)

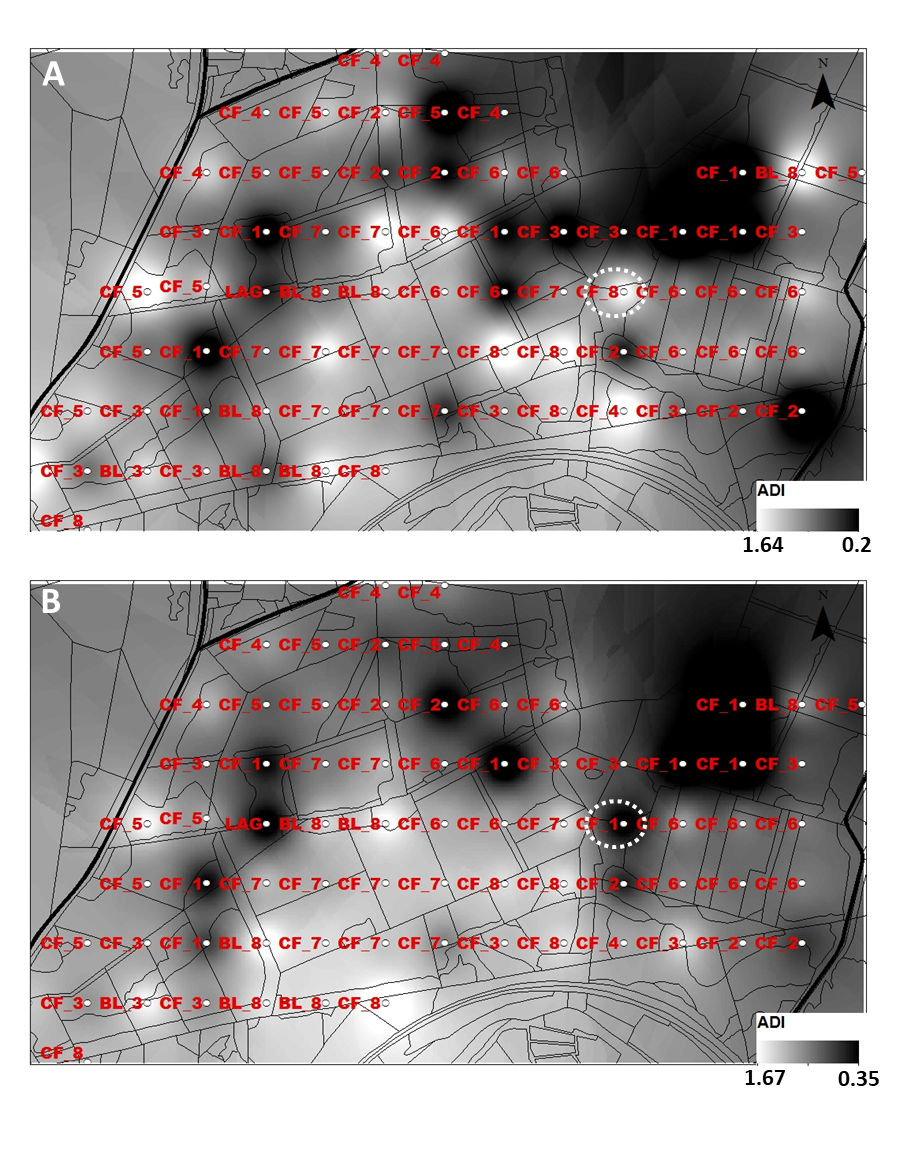

Supplement: S1 Fig — A) 2014 data. B) 2015 data. Darker areas indicate lower acoustic diversity. Site labels are those used in CCA analysis and a key can be found in Table 1 in main body of text. These maps highlight how the acoustic diversity of the area displayed a similar pattern in both years. The dotted circular line (in both maps) shows site 63, which was felled between sampling years and so displayed major changes in the soundscape. These maps highlight the potential for using such sound-mapping techniques for monitoring change between years. Further research into selecting appropriate resolutions in different habitats is key to optimising performance of such tools. (TIF) [file pone.0189843.s002.tif]
